# Supplementary material for: Treatment with Oral Ondansetron for Ultramarathon-Associated Nausea: The TOO FUN Study
Source: Sports (Basel). 2021 Mar 3;9(3):35. doi: 10.3390/sports9030035 (PMC8001581; doi:10.3390/sports9030035)
Supplement: Supplementary file 1 [file sports-09-00035-s001.pdf]

Tahoe Rim Nausea and Vomiting survey

Consent Letter to Participate

Dear Tahoe Rim Trail Endurance Race runner,

You were invited to take part in a research study examining treatment of nausea and vomiting for runners during an ultra marathon.

Dr. Andrew Pasternak, who is also the medical director for the Tahoe Rim Trail Endurance Race, is conducting this study.

Background Information: According to our records, we show that you were given a medication called Zofran (Ondansetron) or a placebo to tae during the Tahoe Rim races in an effort to combat nausea and/or vomiting. While the medication is used routinely for this, there is very little data about how well it works for ultra marathoners. The goal of this study is to see how effective the medication is for nausea and/or vomiting. Your participation in the survey is confidential.

Procedures: If you agree to this questionaire, you will be asked to:

Answer 15 questions through an online survey that will take about 5 minutes to complete.

Examples of some the questions you will be asked include:

-Did you finish the race?
-Did the medications help your symptoms?

Voluntary Nature of the Study: Your participation in this study is voluntary. This means that everyone will respect your decision of whether or not you want to be in the study. If you decide to join the study now, you can still change your mind during the study. If you feel stressed during the study, you may stop at any time. You may skip any questions that you feel are too personal.

Risks and Benefits of being in the Study: There are no foreseeable risks associated with this study. The associated benefit to you, for taking part in this study, is that you are providing information that will be used to help guide runners as to the best way to treat nausea and vomiting.
Payment: There is no compensation for participating in the study.

Privacy: Any information you provide will be kept confidential. The researcher will not collect or use your personal information for any purposes outside of this research project. Data obtained will not include personal information and will be locked in a secured file. The researcher will preserve the file in accordance with IRB requirements and will shred after meeting the retention requirements. Data will be kept for a period of at least 5 years, as required by the university.

Contacts and Questions: You may ask any questions you have now. Or if you have questions later, you may contact the researcher via email at avpiv711@sbcglobal.net or 775.853.9394. If you want to

talk privately about your rights as a participant, you can email Nancy Moody at the University of Nevada Research Integrity Office at nmoody@unr.edu

In order to protect your privacy signatures are not being collected. Completion and submission of the survey implies that you have read the information in this form and consent to take part in the research.

Please keep this consent form for your records or future reference.

To begin the survey click on the link below:

1. Your Name?

* 2. 2017 Tahoe Rim Trail Race number

3. How many ultra marathons have you completed (any race 50K or more)

- This is my first ultramarathon
- 2-5
- 6-10
- 11-15
- 16-20
- More than 20

4. In 2018, other than the TRT endurance races how many ultra marathons have you completed (any race 50K or more)

- This is my first ultramarathon
- 1-2
- 3-5
- 6-8
- 9-10
- more than 11

5. Did you finish your race at the 2017 Tahoe Rim Race?

- Yes
- No

6. Did you take the medications given to you by the research team during the race for any nausea or vomiting ?

- Yes
- No

7. If you took the medications, at approximately what mile did you take the medications given to you? If you took the medications more than once, enter each point where you took the medications.

Mile that you took the first dose of medications ______

Mile that you took the second dose of medications ______

Mile that you took the third dose of medications ______

8. If you took the medication did you take it for:

- ◯ Nausea only
- ◯ Nausea and vomiting
- ◯ Vomiting only

9. If you didn't finish the race, did you not finish due to nausea, vomiting or other gastrointestinal problems?

- ◯ Yes
- ◯ No
- ◯ N/A- I finished the race

10. Do you feel the medication you were given helped your symptoms?

- ◯ Yes
- ◯ No
- ◯ Not sure

11. On a scale from 1-100, where 1 means the medication didn't help at all and 100 means the medications completely got rid of your symptoms, how much did the medication you were given help you?

0 ———————————————— 100 [ ]

12. To the best that you can remember, on a scale from 1-100 with 1 being no symptoms and 100 being severe symptoms, how bad was your nausea/vomiting approximately 1 hour after you took the medication?

0 ———————————————— 100 [ ]

13. To the best that you can remember, on a scale from 1-100 with 1 being no symptoms and 100 being severe symptoms, how bad was your nausea and vomiting BEFORE you took the medication?

0 100

14. To the best that you can remember, on a scale from 1-100 with 1 being no symptoms and 100 being severe symptoms, how bad was your nausea/vomiting when you finished the race or dropped out of the race?

0 100

15. Do you think that you had any side effects from the medication you were given?

- Yes
- No

16. What side effects do you think you had?

17. Do you experience nausea and/or vomiting frequently during runing races?

- Yes
- NO

18. Have you every had to drop out of a race due to nausea, vomiting or gastrointestinal issues in the past

- Yes
- No

19. Have you seen a healthcare provider for exercise related nausea and vomiting in the last 2 years?

- Yes
- No

20. Did you take any of your OWN medications, supplements or other substances for nausea and/or vomiting during the 2016 Tahoe Rim Trail Races?

- Yes
- No

21. Did you have to receive medical care at any of the medical aid stations during the race (Tunnel Creek, Diamond Peak or Start-Finish areas)

- ☐ Yes
- ☐ No

22. Over the past year, approximately how many miles per week did you run?

____

23. In your training leading up to the Tahoe Rim Trail race, how many miles did you run in your maximum training week?

____

24. In your training leading up to the Tahoe Rim Trail race, what was your longest single run?

____

25. If you took the medications, do you you think you took the placebo or the active medication (ondansetron aka zofran)

- ○ Placebo
- ○ Active medication (ondansetron aka zofran)
